# A pilot trial of repetitive transcranial magnetic stimulation of the dorsomedial prefrontal cortex in anorexia nervosa: resting fMRI correlates of response

**DOI:** 10.1186/s40337-021-00411-x

**Published:** 2021-04-17

**Authors:** D. Blake Woodside, Katharine Dunlop, Charlene Sathi, Eileen Lam, Brigitte McDonald, Jonathan Downar

**Affiliations:** 1 Program for Eating Disorders, University Health Network, Toronto, Canada; 2 Centre for Mental Health, University Health Network, Toronto, Canada; 3 Department of Psychiatry, Faculty of Medicine, University of Toronto, Toronto, Canada; 4 Institute of Medical Science, University of Toronto, Toronto, Canada; 5 Feil Family Brain and Mind Research Institute, Weill Cornell Medicine, New York, USA; 6 MRI-Guided rTMS Clinic, University Health Network, Toronto, Canada; 7 Krembil Research Institute, University Health Network, Toronto, Canada

**Keywords:** Anorexia nervosa, R-TMS, fMRI, Anorexia, Treatment, Neuromodulation

## Abstract

**Background:**

Patients with anorexia nervosa (AN) face severe and chronic illness with high mortality rates, despite our best currently available conventional treatments. Repetitive transcranial magnetic stimulation (rTMS) has shown increasing efficacy in treatment-refractory cases across a variety of psychiatric disorders comorbid with AN, including major depression, Obsessive Compulsive Disorder (OCD), and Post traumatic Stress Disorder (PTSD). However, to date few studies have examined the effects of a course of rTMS on AN pathology itself.

**Methods:**

Nineteen patients with AN underwent a 20–30 session open-label course of dorsomedial prefrontal rTMS for comorbid Major Depressive Disorder (MDD) ± PTSD. Resting-state functional MRI was acquired at baseline in 16/19 patients.

**Results:**

Following treatment, significant improvements were seen in core AN pathology on the EDE global scale, and to a lesser extent on the shape and weight concerns subscales. Significant improvements in comorbid anxiety, and to a lesser extent depression, also ensued. The greatest improvements were seen in patients with lower baseline functional connectivity from the dorsomedial prefrontal cortex (DMPFC) target to regions in the right frontal pole and left angular gyrus.

**Conclusions:**

Despite the limited size of this preliminary, open-label study, the results suggest that rTMS is safe in AN, and may be useful in addressing some core domains of AN pathology. Other targets may also be worth studying in this population, in future sham-controlled trials with larger sample sizes.

**Trial registration:**

Trial registration ClinicalTrials.gov NCT04409704.

Registered May 282,020. Retrospectively registered.

## Plain English summary

Anorexia nervosa is a serious illness that is difficult to treat. New treatments are urgently needed. This paper describes a preliminary study of a potential new treatment for Anorexia Nervosa, transcranial magnetic stimulation. In this treatment a magnetic field is generated and applied from outside the skull to affect specific areas of the brain thought to be involved in Anorexia nervosa. The results of this preliminary trial, conducted with 19 subjects over 6 weeks, showed some improvements in the way people with Anorexia nervosa think about weight, shape and their eating. The benefits were associated with areas of the brain involved in decision making and in regulating emotions. These changes did not appear to be related to improvements in other conditions, such as depression.

## Introduction

Anorexia Nervosa (AN) is a serious medical condition with a chronic, treatment-resistant course as well as substantial rates of mortality. While perhaps half of those affected will eventually respond to conventional treatments, many do not, and either develop a chronic intractable course or die from their condition. The evidence base for conventional treatments is weak, and the development of new treatments for AN has been slow, with few truly innovative new treatments over the last several decades. Given the severity of the condition, it is urgent that new approaches to treatment be examined.

Repetitive transcranial magnetic stimulation (rTMS) is an emerging neuromodulation treatment currently approved in major depression and obsessive-compulsive disorder, and used investigationally in a variety of other psychiatric conditions. The most common target of stimulation in most conditions is the dorsolateral prefrontal cortex. However, as the technology has matured, new stimulatory targets have become available, including the dorsomedial prefrontal cortex (DMPFC) [1]. In addition, the emergence of functional imaging, such as functional magnetic resonance imaging (fMRI) has allowed for visualization of changes in brain activity in response to various treatments, and clinical improvement can be correlated to changes in such activity over the course of treatment.

Our group has recently examined the use of neuronavigated DMPFC-rTMS supplemented by fMRI in a number of conditions relevant to eating disorders. DMPFC-rTMS has achieved significant symptom improvement in Bulimia Nervosa (BN, [11]), Obsessive-Compulsive Disorder [10], and Post-Traumatic Stress Disorder (PTSD, [20]). In the first two studies, we were able to correlate changes in key symptoms to alterations in relevant brain circuitry function seen on resting-state fMRI. However, to date, no studies have reported clinical or neuroimaging findings during DMPFC-rTMS for AN.

To date, there is an extremely small literature on the use of rTMS in AN. All of this literature involves stimulation of the dorsolateral prefrontal cortex (DLPFC). Van den Eynde et al. [19] reported on 10 subjects who received a single session of DLPFC rTMS and noted some subjective reductions in feeling ‘fat’ or ‘full’, and some reduction in anxiety. McClelland et al. [14] reported on two cases of severe AN who received approximately 20 sessions of DLPFC rTMS, noting some improvements in EDE symptoms and mood, persisting out to 1 month post treatment. One patient reported decreases in the frequency of bingeing and vomiting. This team then reported on the results of a single session, double blind DLPFC protocol on 49 AN subjects [15] showing non-significant improvements in some AN symptoms amongst those who received active treatment. Finally, Choudhary et al. [6] reported on a single individual with severe AN who received 21 sessions of DLPFC rTMS, and while the authors reported that the patient improved across a wide variety of domains, no quantitative data were presented aside from BMI scores.

The current study extends some of the above findings using a new target for stimulation, the dorsomedial prefrontal cortex (DMPFC), in an open-label sample of 19 patients with AN. We report the effects of this intervention on AN psychopathology, as well as associated changes in brain activity on resting-state fMRI that may correlate with any changes in AN psychopathology..

## Methods

### Demographic and psychometric data

Between May 2012 and July 2017, 19 female patients with AN (ages 21–56, mean 31.2 ± SD 9.8; BMI 14.5–18.5, mean 16.4 ± SD 1.3) underwent a course of DMPFC-rTMS at the UHN Magnetic Resonance Imaging (MRI)-Guided rTMS Clinic, in each case for an indications other than the AN per se (i.e., comorbid major depression and/or PTSD). The diagnosis of AN was based on a full clinical interview conducted by a Canadian Royal College-certified psychiatrist (authors DBW, JD) according to the Diagnostic and Statistical Manual fourth edition (DSM-IV) criteria.

### rTMS treatment parameters

All patients underwent an initial course of 20 sessions of bilateral DMPFC-rTMS, administered once daily on weekdays over 4 weeks; extension to 30 sessions was offered to those showing partial clinical improvement (mean course length, 22.6 sessions). Stimulation followed the same procedures we have reported previously for DMPFC-rTMS in MDD [1]. To summarize, treatment sessions employed a MagPro R30 stimulator and Cool-DB80 coil positioned laterally to the midline at 25% of the nasion-inion distance, with stimulation of the left then right hemisphere accomplished by orienting the coil handle to the right then to left during the two runs of stimulation on each daily session. All subjects received at least 20 sessions of 10 Hz stimulation.

### Outcome measures

Clinical and psychometric data was collected at the initiation and conclusion of treatment. Subjects were administered the Eating Disorders Examination (EDE 16.0D, [7]), the Beck Depression Inventory (BDI, [3]), the Hamilton Depression Inventory (HAM-D, [13]), the Beck Anxiety Inventory (BAI, [2]) and the Difficulties with Emotional Regulation Scale (DERS, [12]). The EDE and HAM-D were administered by trained assessors. Significance testing for changes from pre- to post-treatment was performed via paired t-tests on each measure.

### fMRI acquisition and analysis

For 16 participants, a baseline (pre-treatment) structural and resting-state functional fMRI was available to investigate resting-state functional connectivity correlates to Global EDE improvement. Subjects underwent scanning the week prior to rTMS treatment in a 3 T GE Signa HDx scanner equipped with an 8-channel phased-array head coil. A T1-weighted anatomical (TE(time to echo) = 12 ms; TI = 300 ms; flip-angle = 20 degrees; 116 sagittal slices; slice thickness = 1.5 mm, no gap, 256 × 256 matrix, FOV(field of view) = 240 mm) and a 10-min eyes-open resting-state functional MRI scan (TE = 30 ms; TR = 2000 ms; flip-angle = 85 degrees; 5 mm thick axial slices, no gap, 64 × 64 matrix, FOV = 220 mm, 300 volumes) were collected.

Resting-state fMRI data was preprocessed using the Conn functional connectivity toolbox (version 16b, http://www.nitrc.org/projects/conn/) under Matlab version 8.5.0 and implementing functions from SPM12 (Statistical Parametric Mapping, Wellcome Trust Centre for Neuroimaging, London, UK; http://www.fil.ion.ucl.ac.uk/spm). The following preprocessing steps were taken: removal of the first 5 volumes to account for image stabilization; functional realignment, unwarping and centering; slice-timing correction; structural centering, segmentation and normalization; ART-based (Artifact detection tool) motion-parameter scrubbing, functional smoothing (6 mm FWHM:full width at half maximum); aCompCor-based (anatomical component correction) removal of physiological noise [4]; linear regression of demeaned confounding motion effects; band-pass filtering (0.008–0.09 Hz), and linear de-trending.

Analysis focused on two regions of interest (ROI), as utilized in our previous studies [10, 18]. Briefly, a bilateral DMPFC and dorsal anterior cingulate cortex (dACC) ROI were defined a priori from a parcellation atlas by Craddock et al. [8]. These ROIs were selected based on their proximity to the stimulation target and for consistency with our previous fMRI studies of DMPFC-rTMS. The time series of each ROI was used as a regressor to generate statistical parametric maps at the individual-level. The resultant statistical parametric maps were subsequently used in a higher-level mixed-effects general linear model (GLM). For both GLMs, ROI resting-state functional connectivity was correlated with de-meaned global-EDE percent improvement from pre- to the first follow-up visit at 0–4 weeks post treatment, with age and Body Mass index (BMI) included as covariates. If the first follow-up data was unavailable, end-of-treatment data was used (*n* = 4). Group-level analyses were thresholded at false-discovery rate corrected cluster-*p* < 0.05 and a height threshold of *p* < 0.001.

## Results

### Clinical measures

Results are presented in Table 1. Average BMI at treatment was 16.4, and declined slightly to 16.3 at the end of treatment. The EDE global scale showed significant improvement from pre- to post-treatment (*p* < 0.010) even after Bonferroni correction for the 5 clinical scales examined. Exploratory analyses of the EDE subscales, using an uncorrected threshold of *p* < 0.05, showed significant improvements in shape concerns (*p* = 0.042) and weight concerns (*p* = 0.024), and a trend toward improvement in eating concerns (*p* = 0.054). There was also a significant improvement in anxiety as measured on the BAI (*p* < 0.001), which remained significant after Bonferroni correction across the 5 scales examined. The BDI also showed improvement (*p* = 0.041), and the HAMD a trend toward improvement (*p* = 0.066), although these did not survive correction for multiple comparisons.

**Table 1:** Pschometric measures of effect for DMPFC-rTMS in AN

Variable	Pre-treatment	Post-treatment	***p***-value
***EDE global***	***4.12***	***3.35***	.010**
EDE restraint	3.65	3.21	.095
EDE eating concerns	3.92	3.52	.054
EDE shape concerns	4.64	3.63	.042*
EDE weight concerns	4.23	3.35	.024*
BDI	36.16	30.24	.041*
HAM-D	15.50	14.28	.066
BAI	23.43	12.87	<.001*
DERS	115.33	117.00	.594
**Age at start of treatment**	21–56, mean 31.2 ± SD 9.8		
**Body Mass Index**	14.5–18.5, mean 16.4 ± SD 1.3		

Significant improvements were seen in the EDE global score and the BAI, after Bonferroni correction for multiple comparisons (** = *p* < 0.05, corrected). Considering uncorrected *p*-values in an exploratory fashion, the EDE subscales for shape and weight concerns as well as the BDI showed significant improvement (* = *p* < 0.05, uncorrected)

### fMRI results

#### dACC resting-state connectivity

Baseline resting-state functional connectivity to the dACC ROI did not significantly correlate with global EDE score improvement for any area of the brain.

#### DMPFC resting-state connectivity

**Fig. 1:**
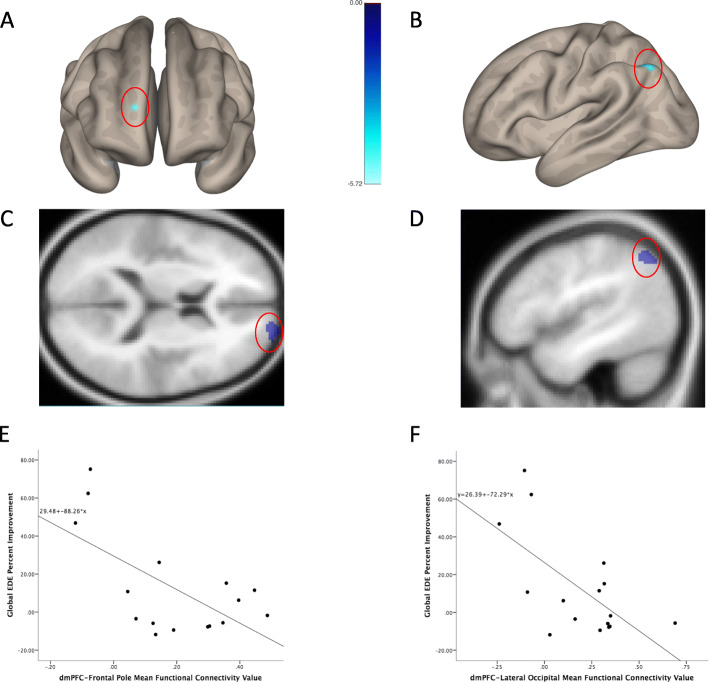
Brain regions where baseline functional connectivity to DMPFC predicted subsequent improvement in EDE global score. For regions in the right frontal pole (**a**, **c**) and left angular gyrus (**b**, **d**), lower baseline connectivity to the DMPFC predicted greater subsequent improvement following the course of DMPFC-RTMS (**e**, **f**)

Baseline resting-state functional connectivity to the DMPFC ROI revealed two significant clusters that negatively correlated with percent global EDE score improvement. Lower pre-treatment resting-state functional connectivity from the DMPFC to the right frontal pole and left angular gyrus significantly correlated with symptomatic improvement (Table 2; Fig. 1). Mean parameter estimates extracted from each cluster also significantly correlated with percent EDE improvement (Frontal pole/EDE improvement *r* = − 0.63, *p* = 0.009; Angular gyrus/EDE improvement *r* = − 0.64; *p* = 0.008).

**Table 2:** Regions showing significant correlation between baseline DMPFC resting-state functional connectivity and subsequent improvement in EDE global score

MNI			# Voxels	cluster p-FDR	Hemi	Region
x	y	z				
18	64	16	100	0.048	R	Frontal Pole
− 46	− 62	44	90	0.048	L	Sup. Lat. Occipital Cortex, Angular Gyrus

*Abbreviations: Hemi* Hemisphere; *FDR* False Discovery Rate; *L* Left; *Lat* Lateral; *MNI* Montreal Neurological Institute; *R* Right; *Sup* Superior

## Discussion

This study is the first examining the use of DMPFC-rTMS in AN, and only the second study that we are aware of that has provided subjects with a full course of treatment (i.e., ≥20 sessions). It is the first study of rTMS in AN with associated functional imaging.

Regarding clinical outcomes, the psychometric findings showing some modest reductions in core AN psychopathology are of interest, especially as the improvements in these core domains of pathology were seen over a rapid timeframe of 4–6 weeks, and there was no adjunctive eating disorder treatment (psychotherapy or medication adjustments) being received by subjects. The lack of change in weight over the 4–6 week course is not unexpected, given the short duration of the treatment. The accompanying improvements in mood and anxiety are consistent with observations we have previously reported in other studies of DMPFC-rTMS in eating disorders [10, 11, 20], and suggestive of a transdiagnostic mechanism of effect for DMPFC-rTMS, as proposed elsewhere [9].

The neuroimaging findings offer some suggestion that patients who show EDE improvement may have distinctive patterns of network connectivity centering on the stimulation site. For example, the frontal polar region identified in the present study (Fig. 1) has been previously shown in healthy controls to be functionally coactive with the ‘salience network’, a network of regions that includes the DMPFC and which plays a major role in cognitive control and impulse control [16]. In the present study, patients lacking this connectivity (perhaps in reflection of a lesser capacity for cognitive control over perseverance or negative self-referential thinking) showed the greatest EDE improvement in DMPFC-rTMS. Notably, in healthy controls, DMPFC-rTMS has been shown to enhance the capacity for impulse control on the delay discounting task [5]. The possibility that DMPFC-rTMS may be particularly well suited to a subpopulation of AN patients with comorbid impulsivity or emotional dysregulation bears further investigation in future studies.

A number of limitations to the present study bear acknowledgement. First, this preliminary study had a small sample size, which may have led to underpowering to detect therapeutic effects, and may also have made it difficult to identify or characterize DMPFC-rTMS responsive subgroups of patients, as above. Second, the study used an open-label design, precluding assessment of the contribution of non-specific/placebo effects in the therapeutic response. Mitigating this point, the patients in this question were presenting for treatment of major depression or PTSD, without expectation of changes in core AN pathology as assessed on the EDE. Nonetheless, follow-up studies to this preliminary pilot work should employ a randomized, sham-controlled design. Finally, as this was primarily a clinical sample, only baseline and not follow-up MRIs were available; this precluded the assessment of changes in DMPFC connectivity that might illuminate the mechanisms of therapeutic effect. As we have previously reported that changes in DMPFC-striatal-thalamic connectivity are associated with improvement in MDD, BN, and OCD symptoms [10, 11, 18], it would be interesting to determine whether similar changes accompany improvement in core AN pathology on the EDE.

## Conclusions

This preliminary case series suggests that DMPFC-rTMS may yield improvements in some elements of AN pathology. Given that previous work suggesting that DMPFC-rTMS enhances impulse control [5], further investigation may clarify whether the treatment is best suited to a subgroup of AN patients with deficits in salience network integrity and impulsivity of thought and behavior. It may also be worth investigating whether a more recently developed rTMS target in the right orbitofrontal cortex, originally showing benefits in OCD [17], might address different elements of AN pathology, such as obsessionality, rigidity, or perseveration. Targeting the neural substrates of AN pathology non-invasively with rTMS may eventually provide an important adjuvant to conventional treatments, in cases where such treatments have failed to achieve meaningful improvement.

## Data Availability

The dataset used in this publication can be obtained from the corresponding author on reasonable request.

## Abbreviations

AN: Anorexia Nervosa
rTMS: Repetitive transcranial magnetic stimulation
OCD: Obsessive Compulsive Disorder
PTSD: Post Traumatic Stress Disorder
MDD: Major Depressive Disorder
DMPFC: Dorsomedial Prefrontal Cortex
rMRI: Functional magnetic resonance imaging
BN: Bulimia Nervosa
DLPFC: Dorsolateral Prefrontal Cortex
MRI: Magnetic resonance imaging
DSM: Diagnostic and Statistical Manual
EDE: Eating Disorders Examination
BDI: Beck Depression Inventory
HAM: Hamilton Depression Inventory
BAI: Beck Anxiety Inventory
DERS: Difficulties with Emotional Regulation Scale
ROI: Region of interest
TE: Time to echo
SPM: Statistical Parametric Mapping
ART: Artifact Detection Tool
FWHM: Full width half medium
aComp-Cor: Anatomical Component Correction
dACC: Dorsal anterior cingulate cortex
GLM: General Linear Model
BMI: Body Mass Index
